# Frequent Video Game Players Resist Perceptual Interference

**DOI:** 10.1371/journal.pone.0120011

**Published:** 2015-03-25

**Authors:** Aaron V. Berard, Matthew S. Cain, Takeo Watanabe, Yuka Sasaki

**Affiliations:** Laboratory for Cognitive and Perceptual Learning, Brown University, Department of Cognitive, Linguistic, and Psychological Sciences, Providence, Rhode Island, United States of America; Nothwestern University, UNITED STATES

## Abstract

Playing certain types of video games for a long time can improve a wide range of mental processes, from visual acuity to cognitive control. Frequent gamers have also displayed generalized improvements in perceptual learning. In the Texture Discrimination Task (TDT), a widely used perceptual learning paradigm, participants report the orientation of a target embedded in a field of lines and demonstrate robust over-night improvement. However, changing the orientation of the background lines midway through TDT training interferes with overnight improvements in overall performance on TDT. Interestingly, prior research has suggested that this effect will not occur if a one-hour break is allowed in between the changes. These results have suggested that after training is over, it may take some time for learning to become stabilized and resilient against interference. Here, we tested whether frequent gamers have faster stabilization of perceptual learning compared to non-gamers and examined the effect of daily video game playing on interference of training of TDT with one background orientation on perceptual learning of TDT with a different background orientation. As a result, we found that non-gamers showed overnight performance improvement only on one background orientation, replicating previous results with the interference in TDT. In contrast, frequent gamers demonstrated overnight improvements in performance with both background orientations, suggesting that they are better able to overcome interference in perceptual learning. This resistance to interference suggests that video game playing not only enhances the amplitude and speed of perceptual learning but also leads to faster and/or more robust stabilization of perceptual learning.

## Introduction

Every day, more of our society is exposed to rapid video stimulation and virtual environments, ranging from television and movies to interactive games requiring active and vigilant participation. The frequency of exposure is becoming more prevalent in today’s youth as well as among the general population, raising questions of how such activity affects our brain. In the past decade, studies have been conducted examining the differences in visual skill and function with frequent gamers compared to non-gamers. There exists a plethora of viable evidence suggesting differences caused by frequent exposure to video games. This study focuses on the lasting effects of video game exposure and how this activity could influence visual learning.

Earlier studies produced robust effects concerning the effect of frequent video game playing on visual skills and attentional abilities. It has been shown that frequent gamers have enhanced abilities in a variety of visual and attentional skills in comparison to non-gamers. Subjects who frequently played action video games were proficient in processing multiple distractors, quickly identifying visual targets, stretching visual attention over a wide eccentricity range, and resisting the “attentional blink” effect [1]. These findings suggest that frequent gamers have enhanced attentional resources unlike their non-gamer counterparts. In addition, these finding have been repeatedly confirmed [2–7].

Importantly, frequent video gaming seems to affect visual plasticity [8] where performance improvement trends seen with frequent video game playing resemble typical aspects of Visual Perceptual Learning (VPL). VPL is defined as long-term enhanced performance as a result of visual experience [9]. Frequent gamers have demonstrated trends in performance change similar to the core principles of VPL, such as higher contrast sensitivity [10] and better spatial resolution [11]. Thus, frequent video gaming could be considered to be a type of visual perceptual learning (VPL). However, little is known about how video gaming affects temporal dynamics in visual plasticity [8]. In case of VPL, there is an important time course dynamics such as consolidation [12–18]. Consolidation here refers to a progressive post-acquisition stabilization of long-term learning as well as to the learning phase(s) during which such presumed stabilization takes place [16,19,20]. It has been suggested that at least one hour is necessary for newly encoded perceptual learning to be stabilized so that it is not disrupted or interfered with training of another type of perceptual learning. For example, performance improvement in a task can be interfered with following a similar task if the second task takes place within an hour of the first [17,21]. This effect however, can be described in two modes of interference: anterograde and retrograde. Anterograde interference refers to when performance improvement on the second task is disrupted, whereas retrograde refers to disrupted performance on the first task [17].

If frequent gamers have an enhanced capacity and speed in lower visual processing accompanied with more attentional resources, they may show little interference in learning two similar tasks within a short time window. To address this question, we conducted an interference paradigm that we have developed earlier [17] in a Texture Discrimination Task (TDT) [17,22]. We have found that such an interference effect was not observed with a frequent gamer when trainings of two types of perceptual learning were conducted with no time interval between them. In contrast, these results were not seen in a non-gaming population. Thus, the present results suggest that video game playing not only enhances the capacity and speed of perceptual learning but *also* leads to faster and/or more robust stabilization of perceptual learning.

## Materials and Methods

### Participants

Participants were recruited from the Brown University campus using flier and email contact. All participants who volunteered had normal to corrected vision and were aged between 18–25 years old (mean 19.94, ± 0.45 SEM). This study collected 9 frequent gamers (2 female and 7 males) and 9 non-gamers (8 females and 1 male). The gamers had a mean age of 20.33 (± 0.55 SEM) and the non-gamers had a mean age of 19.56 (± 0.73 SEM). The institutional review board of Brown University approved this study. Subjects gave their written informed consent for their participation after the purpose of procedure of the study was thoroughly described.

Frequent gamers and non-gamers were classified according to a survey inquiring about video game playing habits utilizing questions from similar studies [1,8,11]. Frequent gamers were classified as those who participated in action video game playing (as defined by previous research [1] at least 5 hours a week for a period of 6 months or more continuously (mean 5.64 hours/week, ± 1.88 SE). Non-gamers were classified as those who played less than 1 hour a week in a given 6-month period (mean 0.32 hours/week ± 0.11 SEM). Most frequent gamers and non-gamers were very polarized where frequent gamers played actively and continuously while non-gamers typically did not play any form of video game at all (t(8) = 2.76, p = 0.02).

### Procedures

In the present study, subjects were given a modified version of the TDT paradigm, used in the Yotsumoto and colleagues (2009) study, where subjects were trained on two different TDT backgrounds in immediate succession. This training produced an interference effect where the training from each background interfered with the training from the other. This notion refers back to the concept of consolidation, where a performance improvement would be seen in this paradigm if an hour of rest were allowed in between learning the two backgrounds.

**Fig 1:**
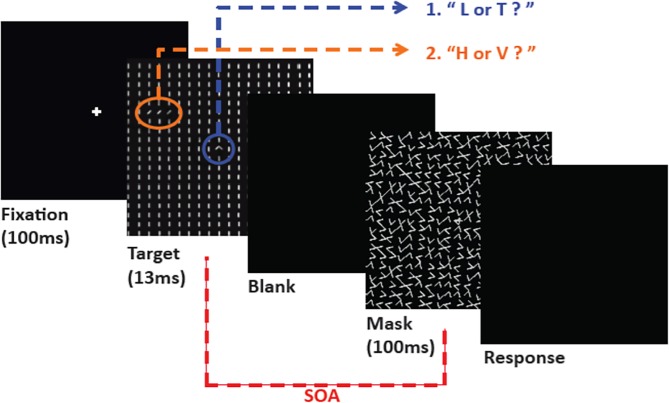
The texture discrimination task (TDT) stimulus. An adapted version of the standard TDT used frequently in VPL experimentation. The first target is highlighted in blue where subjects report either the presence of an L or T, which is designed to hold fixation. This is referred to as the fixation task. The second target (peripheral orientation task) is highlighted in orange and requires the subject to respond with an H or V depending if the targets orientation was horizontal or vertical. The peripheral orientation task is the primary measure of performance in the experiment. Note that the blue and orange circles are provided just for illustrative purposes. They did not appear in the actual experiment. The shorter the SOA, the more difficult the task becomes.

TDT has been originally developed by Karni and Sagi [22] and can be manipulated to form both task-relevant and task-irrelevant signals that impact perceptual training (Fig. 1). The primary goal for the subject is to discriminate an orientation (by responding either with H or V keys for horizontal or vertical) of a target array of oblique lines imbedded in a series of background horizontal or vertical line segments. The target can be presented in any of the four visual quadrants in the subject’s periphery and is referred to as the peripheral orientation task. For the present study specifically, all subjects were trained in the lower left visual quadrant. An additional task is employed as well in order to hold fixation and attention in the center of the stimulus, where subjects are required to report the presence of an L or T at the fixation cross by pressing the corresponding keys. This task is known as the fixation task. TDT can be varied in difficulty through changing the length of Stimulus-to-mask Onset Asynchrony (SOA), which is the time elapsed between the presentation onset of the stimulus and the onset of the mask. If the mask appears more closely following stimulus presentation (small SOA), the task becomes more difficult. After the presentation of the mask, subjects first enter their response to the fixation task followed by their response to the peripheral orientation task.

**Fig 2:**
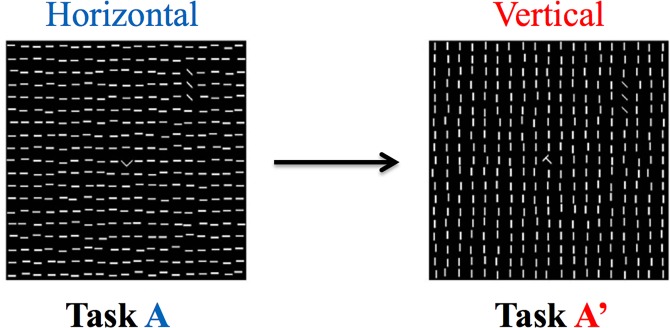
TDT background line orientation changes. An example of using TDT to create the interference in learning Subjects were trained on one background (either horizontal or vertical) and then trained on the opposite background with no resting period in between. Previous research suggests that the immediate switch in background stimulus disrupts learning of one or both backgrounds [17].

The experiment in the present study consisted of two sessions, which spanned for 2 consecutive days. Each session was 24 hours apart and localized to the afternoon in order to avoid any form of circadian effect. One session was divided into 2 parts. The first half of the first session consisted of either all vertical or all horizontal background lines and the second half of the first session consisted of the opposite orientation for the background (Fig. 2). For example, if one subject performed the first half of session 1 with a horizontal background, then their second half would be a vertical background stimulus. The second session (24 hours later) contained the same stimuli parameters as the first session. This procedure was counterbalanced across subject to ensure that the actual stimuli themselves were not confounding the results. The purpose of changing the background line orientation within a session is to cause interference in learning [17], where if this had not occurred one would normally see improvement. The core idea behind this involves the concept of learning consolidation, where not enough time is allowed between different background training for learning to be solidified within the brain’s memory systems [9].

Both frequent gamers and non-gamers were trained with the same number of trials and blocks. In each session, there were 7 blocks, in each of which was conducted with a single SOA with 39 trials. Thus, there were 7 SOAs used and the total number of trials was 273. The SOAs were 180ms, 160ms, 140ms, 120ms, 100ms, 80ms, 60ms, and presented in this order. Throughout the training, the target was presented at a consistent quadrant of the visual field for each subject.

## Results

For analysis, our results have been divided into two separate performance measures, considering both techniques from prior literature [17]. The first measure was the 75% threshold SOA, which was computed as follows. First, we obtained the correct response ratio for the peripheral orientation task computed for each SOA and then fitted this data to a logistic psychometric function. This psychometric curve allows us to easily see performance trends across each SOA and define the threshold for each respective session. The threshold is the point on the psychometric curve that corresponds to the SOA that subjects were able to achieve 75% correct response rate. As noted in our methods, shorter SOAs indicated that the task was more difficult. Thus, the SOA on the curve where subjects had a 75% threshold was the most difficult SOA subjects could handle before dropping below optimal performance rate. If the threshold becomes shorter after training, this indicates that the subject learned the task. The second measure was a simple percent correct for each SOA, which was supplementary to the first threshold measure. While these two measures are correlated, the sensitive aspects may be different.

**Fig 3:**
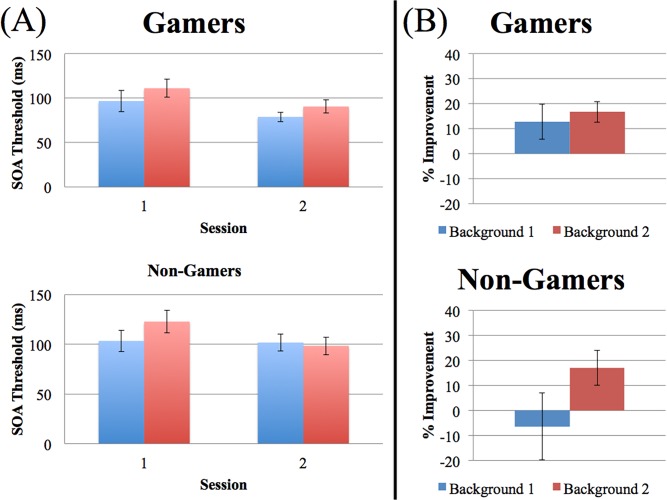
The threshold SOA for both frequent gamers and non-gamers (A). The first background trained is highlighted in blue, whereas the second background trained is highlighted in red. The threshold percent improvement across day 1 and day 2 for both frequent gamers and non-gamers (B). The first trained background is colored in blue whereas the second trained background is colored in red. The frequent gamers and non-gamers differed on background 1 performance change, while not much difference is seen in background 2. Results are shown in standard error.

First, Fig. 3A shows the 75% threshold for each session of the 2-day training in both frequent gamers and non-gamers. In order to confirm learning of the task, a 2x2x2 repeated measures mixed-design ANOVA was conducted on this data, using day, background, and group as factors. The ANOVA revealed a significant main effect of day for both frequent gamers and non-gamers (F(1,17) = 12.840, p = 0.002), suggesting overall performance improvement. Additionally, the main effect of background was also significant (F(1,17) = 4.811, 0 = 0.043) suggesting different trends in performance on each background. The interaction between day, background, and group however, was not significant (F(1,17) = 0.736, p = 0.404), as well as the overall group difference examined through the ANOVA (F(1,17) = 1.430, p = 0.249). Since different trends in performance on each background were suggested through the analyses, we examined the percent change of the threshold data per background across day 1 and day 2 (Fig. 3B). The frequent gamers showed average improvement (10%) for the first background, whereas the non-gamers showed little to no average improvement. This suggests that the retrograde interference occurred with non-gamers, whereas no interference occurred with frequent gamers.

**Fig 4:**
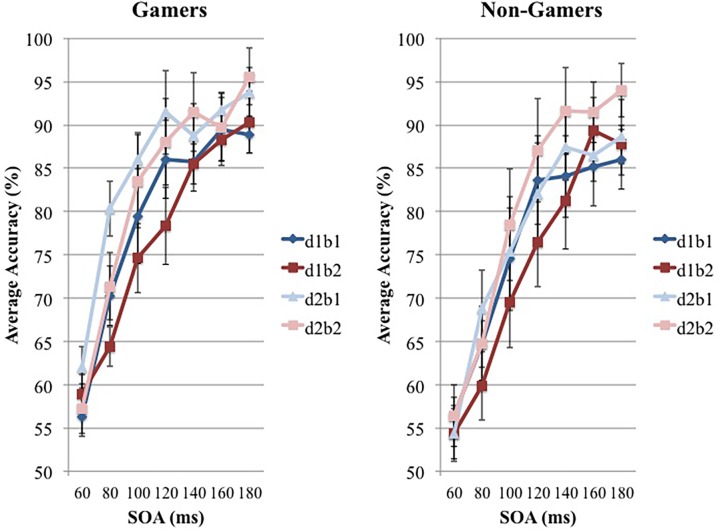
The percent correct for each SOA for both frequent gamers and non-gamers. The blue and light-blue lines represent background 1 whereas the red and light-red lines represent background 2. Darker lines are day 1 and lighter lines are day 2. “d1b1” for example, corresponds to day 1 background 1, and “d1b2” corresponds to day 1 background 2, and so forth. Results are shown in standard error.

Next, Fig. 4 shows the percent correct at each SOA for frequent gamers and non-gamers. Since the effect of retrograde interference should be evident at the performance at background 1, a 2x2 repeated measures ANOVA was conducted on the percent correct data at each presented SOA (Fig. 4) for background 1 with factors day and group yielding a significant group difference (F(1,6) = 10.145, p = 0.008). In order to confirm the effect was specific to background 1, another 2x2 repeated measures ANOVA was conducted on the percent correct data at each presented SOA (Fig. 4) for background 2 with factors day and group. This ANOVA however, did not reveal any significant group difference significant (F(1,6) = 0.378, p = 0.550), suggesting that the presence of retrograde interference with the non-gamers.

## Discussion

The results from the present study suggest that frequent gamers are more resistant to the interference effect seen in a typical population tested on the modified TDT paradigm [17]. Specifically, the non-gamers showed a strong example of retrograde interference, where after learning the second task (background 2), performance improvement on the first task was disrupted. The frequent gamers however, demonstrated this effect to a far lesser degree and produced a positive increase in SOA threshold from day 1 to day 2 with both backgrounds, instead of just the second background as the non-gamers did.

In addition to the retrograde interference findings, an observable difference between performance consistencies can be identified between the frequent gamers and non-gamers. Typically, the frequent gamers exhibited quicker acquisition of the task during training and produced results with less variability than the non-gamers (Fig. 4). This finding however, would be expected due to previous literature [1,10,11], suggesting the enhanced visual abilities of a frequent gaming population, thus denoting more accurate and homogeneous results.

It is important to note the limitations of this study. Specifically, the small number of subjects in each group limits statistical power and could have perhaps concealed additional trends in the data. However, given the significance reached with the current analyses however, the frequent gamers’ resistance to interference may be stronger than originally thought. Additionally, identifying frequent gamers and non-gamers could include some confounding factors. In our study, the frequency in gaming was limited to the past 6 month. Thus, even in the non-gamers in our study, many of them reported playing at least one video game in their life. Also, some reported spending brief periods of time playing video games in their childhood or early adulthood, but not enough to classify them as gamers according to the criteria defined by our questionnaires. It is possible that these people may have experienced changes in visual ability due to brief exposure, since prior research demonstrated enhanced performance on useful-field-of-view, attentional blink, and enumeration tasks after only 10 days of video game training with a non-gaming population [1]. For this reason, we attempted to polarize our subjects by current time spent in gaming as much as possible by measuring gaming activity in the past 6 months in order to establish the largest group difference in gaming experience. Our significant group difference suggests a high degree of polarization between groups (t(8) = 2.76, p = 0.02). The effect of gaming more than 6 months ago on visual processing may be a future study.

Additionally, our efforts with data collection revealed that many frequent gamers happen to be male, thus making it difficult to balance the gender ratio in our participant pool. We attempted to diversify our data collection as much as possible considering this finding, but were only able to a limited extent. Upon investigation however, we could not find any literature supporting sex differences in perceptual learning with interference designs, although there is a recent study that showed the sex difference in the interaction of type of perceptual learning and sleep content [23]. Also, recent data collected in our lab for another project on interference with TDT revealed no statistical difference in behavioral performance trends across males and females [24].

Since this study was conducted over a 24-hour period, we must also consider the confounding factors brought upon by the time subjects spent not in the laboratory, specifically related to sleep. Although encouraged to get a full night’s sleep, it is quite possible that some subjects did not follow instructions and may have been sleep-deprived for the second session. In addition to feeling groggy during testing, such lack of sleep could have left subjects with less time to experience sleep-dependent memory consolidation, which is thought to contribute significantly to storing the information from the TDT training they received the previous day [9]. Specifically, this would result in a disruption of normal sleep architecture possibly allowing for less efficient consolidation, which could lead to less than optimal task performance [14]. Current research however, suggests that performance improvement on TDT can be seen even after only 90 minutes of sleep [12], in which case lack of sleep may not be as confounding as originally thought. It is important to note that this research examined typical TDT paradigms, which did not include methods for inducing the interference effect thought to disrupt consolidation. Thus, it is difficult to conclude that subjects would respond to post-sleep interference TDT testing in a similar manner to post-sleep typical TDT testing. These confounding factors most likely did not occur however, since the results from the statistics showed a strong trend in overall learning with both frequent gamers and non-gamers, which most likely would have been less pronounced had our subjects been sleep-deprived.

With regards to our interest in the role of frequent gaming in learning consolidation, our results allow us to speculate on how frequent gamers may have different solidifying mechanisms operating during sleep and wakefulness. It may be possible that the vast amount of visual training frequent gamers receive over the years could help contribute to honing consolidation mechanisms in the brain, especially for visually developed skills. Essentially, this would mean that over the 24-hour period of time between the experimental sessions, more efficient consolidation mechanisms could have been operating in the frequent gamers compared to the non-gamers, resulting in better overall learning. This could suggest that on top of enhanced perceptual abilities, frequent gaming could help sharpen the mechanisms that allow for the consolidation of visual skills. A new model could be proposed offering insight into how frequent gaming affects not only how we deal with presented information, but also how we retain this information as well.

## Supporting Information

S1 Raw Data Raw data supplementary information. File contains the relevant raw data used to calculate all present figures and statistical operations.
(XLSX)
